# Enchondroma of the scaphoid: a case report

**DOI:** 10.11604/pamj.2015.21.133.6918

**Published:** 2015-06-17

**Authors:** Sbai Mohamed Ali, Benzarti Sofien, Sbei Feten, Bouzaidi Khaled, Maalla Riadh

**Affiliations:** 1 Department of Orthopedic Surgery and Trauma, Nabeul, Tunisia; 2 Department of Radiology, Nabeul, Tunisia; 3 Department of Plastic Surgery, Rabta Hospital, Tunis, Tunisia

**Keywords:** Enchondroma, scaphoid, pathological fracture, bone grafting, carpal bone, wrist

## Abstract

Enchondroma represents a common bone tumor of the hand. The scaphoid is a rare location. We report the case of a scaphoid enchondroma presenting as chronic wrist pain following a relatively minor trauma. The diagnosis was suggested by radiological study and then confirmed by histological study after biopsy. Enucleation and vascularized bone graft with osteosynthesis provided good results. The clinical, radiological and therapeutic aspects of this rare condition are discussed.

## Introduction

Enchondroma is a common benign cartilaginous turnout that grows from the medullary cavity, in particular in the phalanxes of the hand and feet. Carpal bone enchondroma however, is rare; few cases of carpal bone enchondroma have been reported in the literature. We report a rare case of an enchondroma of the scaphoid presenting as chronic wrist pain following a relatively minor trauma.

## Patient and observation

**Figure 1:**
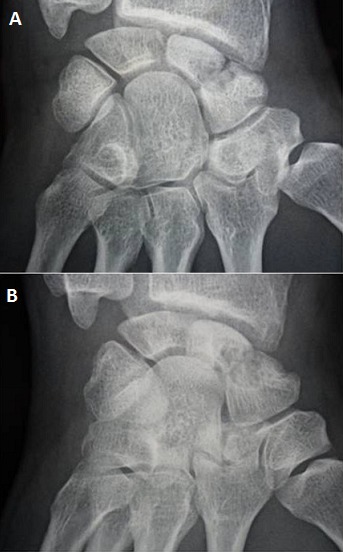
(A) anteroposterior radiograph demonstrate cystic lucency in the middle third of the scaphoid. The speckled calcification in the cyst is suggestive of an enchondroma; (B) Scaphoid view radiograph demonstrate cystic lucency in the middle third of the scaphoid. The speckled calcification in the cyst is suggestive of an enchondroma

**Figure 2:**
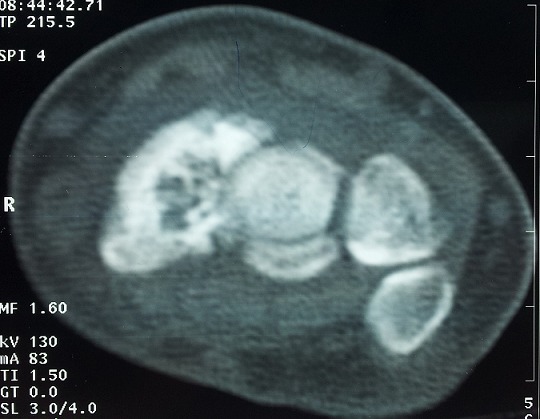
Scan of the wrist providing precise localization of the lesion, revealed the fracture of the scaphoid and the speckled calcification

**Figure 3:**
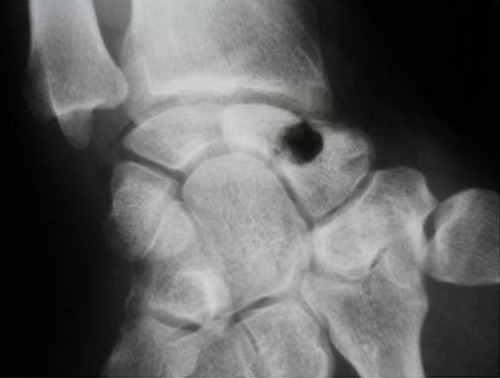
Per operative radiograph demonstrate curettage of the lesion

**Figure 4:**
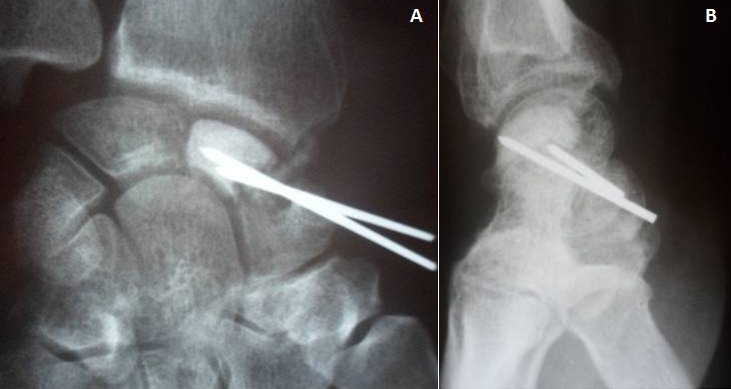
(A) Anteroposterior radiograph shows vascularized bone graft fixed by kirschner wires; (B) Lateral radiograph shows vascularized bone graft fixed by kirschner wires

**Figure 5:**
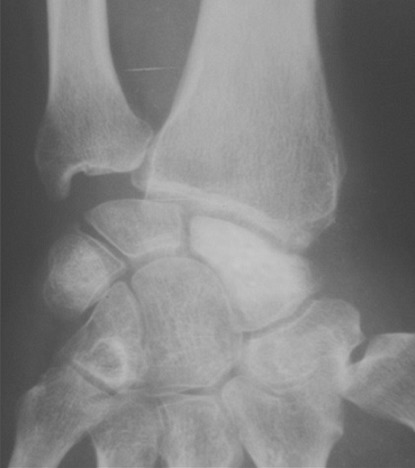
Anteroposterior radiograph at 4-year follow-up demonstrating good integration of the bone graft with no recurrence

A 34-year-old manual worker complained from pain in his right wrist. The symptoms became more severe after strenuous work. There was no reported history of previous major trauma, collagenous or infectious disease. Six months earlier, the patient had experienced sudden pain and tenderness in his right wrist, which was diagnosed as tendinopaty and treated with a splint and a non-steroidal anti-inflammatory drug. Clinical study showed slight swelling of the dorsoradial aspect of the joint, limited and painful motion, and decreased grip strength. Radiographs revealed a fracture through a lucency in the middle third of the scaphoid, the speckled calcifications in the cyst are suggestive of an enchondroma (Figure 1). Scan computed tomography confirms the fracture of scaphoid and provides a precise localization of the lesion and the speckled calcifications. The other bones of the carpus were of homogeneous density and normal in shape (Figure 2). The diagnosis was confirmed by histological study after biopsy. A dorsolateral approach was performed. The lesion was meticulously enucleated then curetted until both proximal and distal surfaces demonstrated diffuse bleeding when the tourniquet was released (Figure 3). The remaining gap was filled with a Zaidenberg vascularized bone graft. Two percutaneous Kirschner wires were placed to fix the grafted scaphoid (Figure 4). The arm was immobilized for 6 weeks in a long cast with the wrist in slight ulnar deviation and the thumb in opposition. Histology showed small fragments of bone and ossifying cartilage in a reactive fibroblastic connective tissue stroma, consistent with an enchondroma. Four years postoperatively, wrist motion was at 60° of flexion and 45° of extension and an arc of 30° for radioulnar deviation. The bone graft was fully incorporated and remodelled without signs of recurrence (Figure 5).

## Discussion

Enchondroma is a benign tumor of hyaline cartilage and represents the second most common benign chondral tumor after osteochondroma. It is assumed that enchondromas arise due to the displacement of embryonic remains of cartilage growth plate in the metaphysis. Regarding the location, around 40-65% of enchondromas are found in the small bones of the hand especially the proximal phalanx [1]. All other locations are rare. The carpus is another rare location of occurrence for enchondromas [2]. Takigawa [3] reported 110 cases of hand enchondromas, only 2 cases were involving the carpal bones (scaphoid, lunate). On the other hand, enchondromas are even uncommon within all carpal lesions. Emecheta [4], reported only three enchondromas in a serie of 86 treated carpal bone lesions. Enchondroma is usually symptom-free, the growth of the associated bone however causes cortical thinning and endosteal resorption, with a high predisposition to pathologic fracture [5, 6]. Scaphoid is the most common localization of the carpal enchondroma, and it is usually minimally or asymptomatic until a pathologic fracture occurs [7–9]. Takka reported 5 cases presenting with pathologic fracture out of 8 cases of scaphoid enchondroma. Malignant transformation of solitary enchondroma is very rare, and it has not been described in the carpus. Although the standard X-ray findings are essentiel in the diagnosis of enchondroma of the carpal bone, the diagnosis of enchondroma may require further investigations such as CT-scan or MRI. MRI is particularly helpful in identifying enchondroma, demonstrated by a low signal intensity on T1 sequences and a high signal intensity on T2 sequences [10]. Treatment aims to preventing recurrence with an appropriate excision of the lesion, and reconstruction of the scaphoid bone with a grafting procedure with minimal morbidity [11, 12]. A vascularized bone graft was selected over conventional grafting methods [8].

## Conclusion

The enchondroma is a well-known benign bone tumor. Its location in the scaphoid bone is exceptional. The pathologic fracture is the most common way of revelation. Diagnosis is suspected on plain radiographs and CT, and is confirmed by the anatomopathologic study. Enucleation and vascularized bone graft with osteosynthesis provides good results.
